# Carbon ion radiotherapy for prostate cancer with bladder invasion

**DOI:** 10.1186/s12894-021-00871-y

**Published:** 2021-08-06

**Authors:** Yuhei Miyasaka, Hidemasa Kawamura, Hiro Sato, Nobuteru Kubo, Tatsuji Mizukami, Hiroshi Matsui, Yoshiyuki Miyazawa, Kazuto Ito, Takashi Nakano, Kazuhiro Suzuki, Tatsuya Ohno

**Affiliations:** 1 Department of Radiation Oncology, Gunma University Graduate School of Medicine, 3-39-22, Showa-machi, Maebashi, Gunma 371–8511 Japan; 2 Gunma University Heavy Ion Medical Center, Maebashi, Japan; 3 Division of Radiation Oncology, Department of Radiology, Faculty of Medicine, Academic Assembly, University of Toyama, Toyama, Japan; 4 Department of Urology, Gunma University Graduate School of Medicine, Maebashi, Japan; 5 Kurosawa Hospital, Takasaki, Japan; 6 Quantum Medical Science Directorate, National Institutes for Quantum and Radiological Science and Technology, Chiba, Japan

**Keywords:** Carbon ion radiotherapy, Prostate cancer, Bladder invasion

## Abstract

**Background:**

The optimal management of clinical T4 (cT4) prostate cancer (PC) is still uncertain. At our institution, carbon ion radiotherapy (CIRT) for nonmetastatic PC, including tumors invading the bladder, has been performed since 2010. Since carbon ion beams provide a sharp dose distribution with minimal penumbra and have biological advantages over photon radiotherapy, CIRT may provide a therapeutic benefit for PC with bladder invasion. Hence, we evaluated CIRT for PC with bladder invasion in terms of the safety and efficacy.

**Methods:**

Between March 2010 and December 2016, a total of 1337 patients with nonmetastatic PC received CIRT at a total dose of 57.6 Gy (RBE) in 16 fractions over 4 weeks. Among them, seven patients who had locally advanced PC with bladder invasion were identified. Long-term androgen-deprivation therapy (ADT) was also administered to these patients. Adverse events were graded according to the Common Terminology Criteria for Adverse Event version 5.0.

**Results:**

At the completion of our study, all the patients with cT4 PC were alive with a median follow-up period of 78 months. Grade 2 acute urinary disorders were observed in only one patient. Regarding late toxicities, only one patient developed grade 2 hematuria and urinary urgency. There was no grade 3 or worse toxicity, and gastrointestinal toxicity was not observed. Six (85.7%) patients had no recurrence or metastasis. One patient had biochemical and local failures 42 and 45 months after CIRT, respectively. However, the recurrent disease has been well controlled by salvage ADT.

**Conclusions:**

Seven patients with locally advanced PC invading the bladder treated with CIRT were evaluated. Our findings seem to suggest positive safety and efficacy profiles for CIRT.

## Background

Localized prostate cancer (PC) is generally treated with radical prostatectomy, external beam radiotherapy (EBRT), and brachytherapy, with or without androgen-deprivation therapy (ADT) [1]. Although favorable clinical outcomes following these treatments are well known, in the case of locally advanced PC invading adjacent structures, that is, clinical T4 (cT4) PC, the prognosis is not satisfactory [2]. A recent study reported that the addition of local therapy, such as surgery and radiotherapy (RT) to systemic therapy, including ADT, provides a survival benefit even for cT4 PC [3]. Therefore, optimization of these local therapies is of importance in the management of cT4 PC.

Carbon ion radiotherapy (CIRT), which is one of the modalities of EBRT initiated at the National Institute of Radiological Sciences in 1994 in Japan, provides a sharp dose distribution with minimal penumbra and has biological advantages due to its high relative biological effectiveness (RBE) in the Bragg Peak, resulting from a high linear energy transfer [4]. At our institution, the Gunma University Heavy Ion Medical Center, CIRT for localized PC, including tumors invading bladder, has been performed since March 2010. Previous studies showed that CIRT for localized PC was a safe and effective treatment [5–9], but these studies did not include cT4 disease. Considering that CIRT has physical and biological advantages over photon radiotherapy, CIRT may provide therapeutic benefits even for the progressive PC. To evaluate this, we retrospectively reviewed patients with locally advanced PC with bladder invasion treated with CIRT.

## Methods

### Patients

Between March 2010 and December 2016, a total of 1337 patients with clinically nonmetastatic PC received CIRT at our institution. All the patients were pathologically diagnosed with adenocarcinoma. All pre-treatment biopsy specimens were re-evaluated by a central pathologist at Gunma University Hospital. Tumor grades were decided according to the modified Gleason grading system proposed by the International Society of Urological Pathology [10]. Urological examination, trans-rectal ultrasonography, computed tomography (CT), magnetic resonance imaging (MRI), and bone scintigraphy were performed for staging. Cystoscopic examination was performed when the diagnosis of bladder invasion and/or the invaded regions was difficult to confirm by radiographic examinations. Assessing these findings, the institutional cancer board with urological oncologists, radiologists, and radiation oncologists participated in diagnosing clinical stages of PC according to the International Union Against Cancer TNM classification (2002). In this study, we evaluated patients who had locally advanced PC with bladder invasion and without invasion to the rectum, pelvic floor muscles, and pelvic wall. Bladder invasion was diagnosed based on cystoscopic findings in principle; in cases when cystoscopy was not performed before ADT and when there were no apparent cystoscopic findings after ADT, MRI findings before ADT were used for the diagnosis. All the treatment plans were approved by the institutional conference before carrying out the actual treatment.

### Carbon ion radiotherapy

CIRT was performed at a total dose of 57.6 Gy (RBE) in 16 fractions over 4 weeks, with a fractional dose of 3.6 Gy (RBE) at four treatment sessions per week. The prescribed dose was according to previous studies on CIRT [7, 8]. Details of CIRT techniques have been previously reported [9]. The patients were positioned in a customized cradle (Moldcare; Alocare, Tokyo, Japan) with a low-temperature thermoplastic sheet (Shellfitter; Kuraray, Co., Ltd., Osaka, Japan). The bladder was filled with 100 mL 0.9% sterile saline, and the rectum was emptied using an enema just before CT simulation. Treatment planning was performed with Xio-N (Elekta, Stockholm, Sweden and Mitsubishi Electric, Tokyo, Japan) using a set of images of 2-mm-thick CT fused with MRI. Clinical target volume (CTV) included whole prostate, proximal seminal vesicle (SV), and bladder wall, which tumors invaded before ADT. For the tumor invading SV, CTV was expanded to include at least the invaded SV. The planning target volume (PTV1) for the initial nine fractions included CTV plus anterior and lateral margins of 10 mm, cranial and caudal margins of 6 mm, posterior margin of 5 mm, and lateral margins to seminal vesicle of 3 mm. The second PTV (PTV2) for the latter seven fractions was generated by cutting the posterior PTV margin in front of the anterior wall of the rectum [7]. Each field was using a spread-out Bragg peak, which was shaped with multi-leaf collimators and compensation bolus for each patient. Three radiation ports were used in the bilateral and anterior directions. At each treatment session using the anterior port, the bladder was filled with 100 mL 0.9% sterile saline.

### Androgen deprivation therapy

ADT was administered to all the patients for a minimum of 24 months. Patients recieved combined androgen blockade therapy (CAB) consisting of luteinizing hormone-releasing hormone (LH-RH) agonist or antagonist, and antiandrogen for at least 5 months before CIRT and during CIRT. After completing CIRT, all the patients recieved an adjuvant LH-RH agonist or antagonist monotherapy.

### Followup and clinical evaluation

All patients were followed up by physical examination and blood test, including PSA and urine test, at 3-month intervals; CT, MRI, bone scintigraphy, and trans-rectal ultrasonography were performed once a year for 5 years. Adverse events (AE) were evaluated according to the Common Terminology Criteria for Adverse Events (CTCAE) version 5.0 [11]. Biochemical failure was defined in accordance with the Radiation Therapy Oncology Group-Association of Therapeutic Radiation Oncology Phoenix Consensus Conference definition [12].

## Results

### Patients’ characteristics

Seven patients who had locally advanced PC with bladder invasion were identified from the medical record. The patients’ characteristics are summarized in Table 1. The median follow-up period was 78 months (range 37–109). The median age at diagnosis was 65 years (range 53–81). The median initial prostate-specific antigen (PSA) level was 32.1 ng/ml (range 7.8–87.0). Three patients (42.9%) had primary Gleason pattern 5. Five patients (71%) had seminal vesicle invasion. Three patients were diagnosed with bladder invasion by cystoscopic findings, while four patients were diagnosed by MRI findings. The median total duration of the ADT was 32 months (range 24–46).

**Table 1:** Summary of the patients’ characteristics.

Patient number	1	2	3	4	5	6	7
Seminal vesicle invasion	+	+	−	+	−	+	+
Gleason score	5 + 4	4 + 3	4 + 3	5 + 4	4 + 5	5 + 4	4 + 5
Positive cores	8/8	10/10	4/8	6/10	8/8	10/12	10/12
Initial PSA (ng/mL)	11.6	87	37.3	32.1	73.7	7.8	9.39
ADT duration before CIRT (months)	12	5	6	6	6	6	12
Total ADT duration (months)	40	24	28	32	25	46	33

*PSA * Prostate-specific antigen, *ADT* Androgen deprivation therapy, *CIRT *Carbon ion radiotherapy

### Clinical outcomes

The clinical courses of the patients with locally advanced PC are summarized in Table 2. All the patients are alive and being followed-up. There was no grade 3 or worse AE. Acute urinary disorder was seen in four patients (#2, #3, #6, #7). One of these patients (#3) needed an alpha-blocker for urinary frequency. As for late toxicity, one patient (#6) complained about urinary urgency; thus, requiring medication. The patient took aspirin and developed hematuria 16 months after receiving CIRT. However, this AE was easily dealt with using a hemostatic agent (carbazochrome sodium sulfonate hydrate) and was never observed again. Two other patients have also taken medication, which increased the risk of bleeding (cilostazol and ethyl icosapentate), but they had no hematuria induced by CIRT. There were no gastrointestinal AE in these seven patients. After the termination of ADT, six patients were tested for serum testosterone, and the recoveries to the standard value were observed. One patient who was not tested for testosterone presented with a slight increase in PSA within the Phoenix definition and thus was considered to recover from the castration status. Only one patient had a recurrence. A patient (#2) had biochemical and local failure 42 and 45 months after CIRT, respectively. The serum testosterone level was 2.28 ng/mL when there was a clinical failure. The recurrent tumor was detected at the original site, and there was no metastatic disease. Salvage CAB was administered to the patient, after which the recurrent disease was undetected on MRI, and serum PSA level monotonically decreased to less than 0.1 ng/ml and remained low thereafter. The other patients have had no evidence of the disease.

**Fig. 1:**
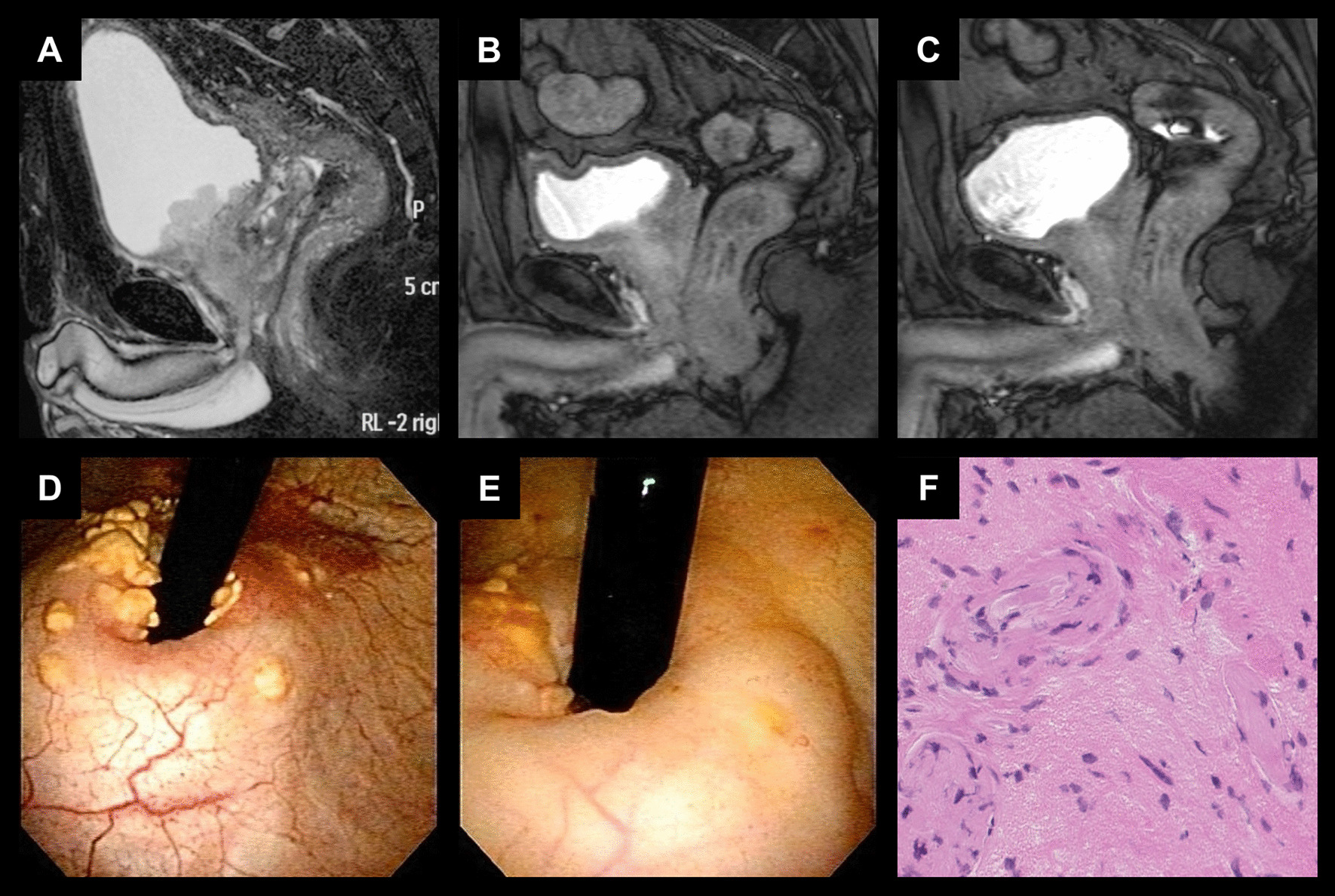
Magnetic resonance imaging (MRI), cystoscopic, and pathological findings of the representative case.  Fat-saturated T2-weighted images **a** before ADT, **b** just before carbon ion radiotherapy (CIRT), and **c** 2 years after CIRT. Cystoscopic findings **d** just before CIRT and **e** one and half a year after CIRT. **f **Hematoxylin-Eosin stain of biopsy sample from the bladder lesion 2 years after CIRT in a high-power field. There were no malignant cells

**Fig. 2:**
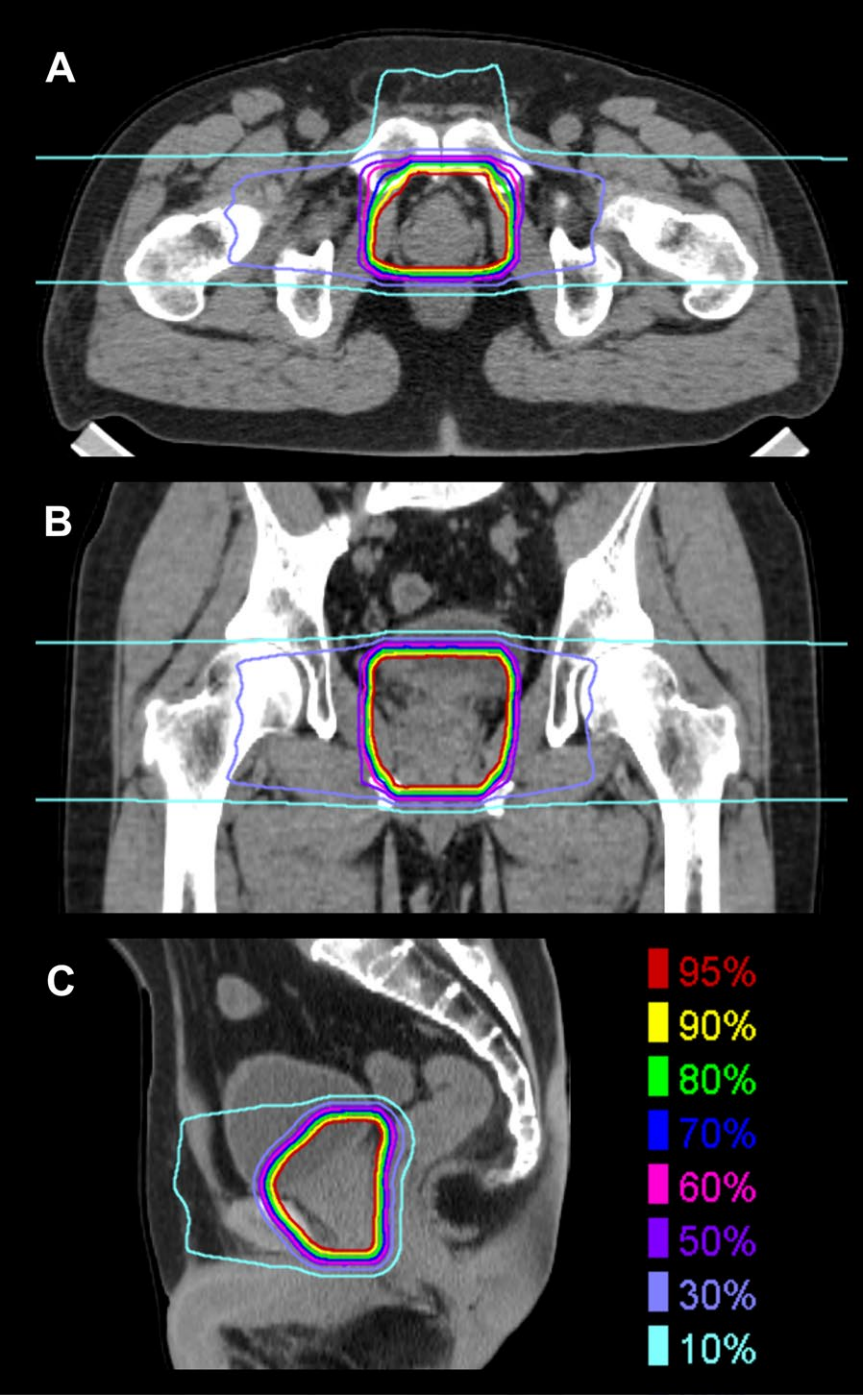
Dose distribution of carbon ion radiotherapy. **a** Axial, **b** coronal, and **c** sagittal images. Highlighted are 95% (red), 90% (yellow), 80% (green), 70% (blue), 60% (pink), 50% (purple), 30% (light purple), and 10% (cyan) isodose curves

**Table 2:** Summary of the clinical course

Patient number	1	2	3	4	5	6	7
Follow-up (months)	109	96	96	78	66	66	37
Alive/ Dead	Alive	Alive	Alive	Alive	Alive	Alive	Alive
Biochemical failure	−	+	−	−	−	−	−
Local failure	−	+	−	−	−	−	−
Distant failure	−	−	−	−	−	−	−
*Acute toxicity (max grade)*							
Genitourinary	0	1	2	0	0	1	1
Gastrointestinal	0	0	0	0	0	0	0
*Late toxicity (max grade)*							
Genitourinary	0	0	1	0	0	2	1
Gastrointestinal	0	0	0	0	0	0	0

### A representative case: Patient #1

In July of 20XX, a Japanese man in his 50 s diagnosed with PC was referred to our institution since he desired to receive CIRT. At that time, approximately 10 months of CAB consisting of leuprorelin acetate and bicalutamide had already reduced serum PSA from 11.8 ng/mL (September of 20XX-1) to < 0.01 ng/mL (August of 20XX), but cystoscopic findings clearly showed tumor invading the bladder neck. The institutional cancer board diagnosed the clinical stage as cT4N0M0 by checking the CT images, MRI, bone scintigraphy, and cystoscopic findings (Fig. 1a, d). The tumor also invaded the right seminal vesicle. Pre-treatment biopsy specimens were reviewed by a central pathologist. Tumor cells were found in all the cores (8/8), and the Gleason score was diagnosed as 5 + 4 = 9.

CIRT was performed at a total dose of 57.6 Gy (RBE) in 16 fractions over 4 weeks from October to November of 20XX. Figure 2 shows the dose distribution. During this treatment period, dermatitis (grade 1) was observed in the irradiated region. There was no other acute toxicity.

After completion of CIRT, bicalutamide was discontinued. Blood and urine tests were performed every 3 months, and CT, MRI, bone scintigraphy, and trans-rectal ultrasonography were performed once a year for 5 years. Chronological changes in MRI findings are shown in Fig. 1b, c. Serum PSA levels were kept under 0.01 ng/mL till ADT was finished. The cystoscopic findings on April of 20XX + 2 showed that the bladder lesion shrank but remained (Fig. 1e). Six months later, as similar findings were found in the cystoscopic examination, a transurethral resection biopsy was performed. The biopsy findings showed urothelial mucosa with xanthogranulomatous lesions and no malignant cells (Fig. 1f). After discussion with urological oncologists in Gunma University Hospital, leuprorelin acetate was discontinued on December of 20XX + 2. Thereafter, the serum PSA level was still well controlled. Approximately 9 years after CIRT, there have been no findings suggesting recurrence or metastasis. No late toxicity was observed.

## Discussion

In the management of cT4 PC, the addition of local therapy to systemic therapy was associated with improved survival compared to systemic therapy alone [3], but the optimal local therapy has not yet been established. We have treated locally advanced PC invading the bladder with CIRT with long-term ADT, expecting that the physical and biological advantages of CIRT over photon RT would yield therapeutic benefits. Thus, we evaluated the safety and efficacy of the CIRT in the current study. To the best of our knowledge, this is the first report describing CIRT with long-term ADT for locally advanced PC with bladder invasion. Our findings showed that none of the seven patients had severe toxicity, and six (85.7%) patients had no recurrence or metastasis with a median follow-up period of 78 months.

There are limited literature on the surgery for cT4 PC [13]. Hajili et al. showed that the prostate cancer-specific survival (PCSS) rates for cT4 PC at 150 months after inductive ADT and subsequent RP were 82%, and 10.3% of the patients had complications requiring surgical intervention [14]. Kumazawa et al. reported cystoprostatectomy followed by immediate hormone therapy for cT4N0M0 disease. In their study, the PCSS rate at 5 years after the surgery was 87.1% [15]. These findings showed relatively favorable survival despite the advanced disease, although it should be noted that these surgical indications were limited to patients with good general conditions.

EBRT, which is a less invasive treatment modality compared to surgery, is also recommended for very high-risk PC, including cT4 disease [1]. Furthermore, intensity-modulated radiotherapy (IMRT) and image-guided radiotherapy enable higher doses to tumors with lower doses to organs at risk, resulting in the lower incidence of AE and improved biochemical relapse-free survival (bRFS) [16]. In addition, EBRT with high-dose-rate brachytherapy boost may improve bRFS [17]. To our knowledge, little is known regarding the outcomes of patients with cT4 PC treated with EBRT, although a clinical trial to analyze whether surgical treatment or EBRT using photons is the better treatment for cT4 PC is undergoing [18].

CIRT, a kind of EBRT, contributes to favorable outcomes, especially in advanced PC. Kasuya et al. reported that the prostate cancer-specific mortality at 5 years after CIRT with long-term ADT was 1.5% for high-risk PC [19]. We previously reported that the 5-year biochemical relapse-free rate of high-risk PC was 92.0% in a single-institutional prospective study [9]. The present study showed that 85.7% (6/7) of the patients had no biochemical failure, and all the patients were alive at the median follow-up period of 78 months. We cannot compare these results with those of EBRT due to the lack of available literature specific to cT4 PC, but when compared with the surgical treatment options, our results seem to be favorable, although we acknowledge that the number of patients included in our study is extremely small.

In general, CIRT is also remarkable for the low incidence of late toxicity because of the superior dose accumulation. We previously demonstrated that 9% of the patients had grade 2 late toxicities after CIRT [9], while Cahlon et al. showed that up to 23% of the patients had grade 2 late toxicities after photon-based IMRT [20]. In CIRT for PC with bladder invasion, the irradiated volume of the bladder was larger than that in PC without bladder invasion, which potentially increases the incidence and severity of urinary toxicity. However, with the careful management of inter-fractional displacements mentioned above, there was only one patient with grade 2 late urinary disorder in the current study; thus, supporting that CIRT is tolerable for patients with locally advanced PC with bladder invasion.

These favorable outcomes of the present study may be due to the physical and biological advantages of CIRT over photon RT, which may have provided therapeutic benefits for locally advanced PC. Although our findings provide only the weakest evidence, we are encouraged to further explore the safety and efficacy of CIRT for PC with bladder invasion in larger cohorts.

The present study has some limitations. As mentioned above, this is a case series report with an extremely small number of patients; thus, some potential sources of bias were not excluded. In addition, the effects of clinical and pathological factors, such as age, initial PSA level, Gleason score, the number of positive cores in biopsy samples, and the duration of ADT, were not evaluated in this study. Larger cohort is required to evaluate these factors.

## Conclusions

In summary, we report seven patients with locally advanced PC with bladder invasion who received CIRT with long-term ADT, with well tolerable toxicity and favorable prognoses. Our study only provides the weakest evidence because of the extremely small study population without control, but CIRT with long-term ADT seems to be a potential treatment option. For more reliable evidence, further studies are required.

## Data Availability

The datasets used and/or analysed during the current study available from the corresponding author on reasonable request.

## Abbreviations

AE: Adverse event
ADT: Androgen-deprivation therapy
bRFS: Biochemical relapse-free survival
CT: Computed tomography
CAB: Combined androgen blockade
CIRT: Carbon ion radiotherapy
CTCAE: The Common Terminology Criteria for Adverse Event
CTV: Clinical target volume
cT4: Clinical T4
EBRT: External beam radiotherapy
IMRT: Intensity-modulated radiotherapy
PC: Prostate cancer
LH-RH: Luteinizing hormone-releasing hormone
MRI: Magnetic resonance imaging
RBE: Relative biological effectiveness
RT: Radiotherapy
PCSS: Prostate cancer-specific survival
PSA: Prostate-specific antigen
PTV: Planning target volume
SV: Seminal vesicle
